# Development of functional human oral mucosal epithelial stem/progenitor cell sheets using a feeder-free and serum-free culture system for ocular surface reconstruction

**DOI:** 10.1038/srep37173

**Published:** 2016-11-14

**Authors:** Takahiro Nakamura, Seiichi Yokoo, Adams J. Bentley, Maho Nagata, Nigel J. Fullwood, Tsutomu Inatomi, Chie Sotozono, Satoru Yamagami, Shigeru Kinoshita

**Affiliations:** 1 Department of Frontier Medical Sciences and Technology for Ophthalmology, Kyoto Prefectural University of Medicine, Kyoto 602-0841, Japan; 2 Department of Ophthalmology, University of Tokyo Graduate School of Medicine, Tokyo 113-8655, Japan; 3 Biomedical and Life Sciences, School of Health and Medicine, Lancaster University, Lancaster, UK; 4 Department of Ophthalmology, Kyoto Prefectural University of Medicine, Kyoto 602-0841, Japan

## Abstract

Ocular surface reconstruction (OSR) using tissue-engineered cultivated oral mucosal epithelial cell sheets (COMECS) is a promising newly developed treatment for patients with severe ocular surface disease. Until now, this technique has used exogenic and undefined components such as mouse-derived 3T3 feeder cells and fetal bovine serum. To minimize associated risks of zoonotic infection or transmission of unknown pathogens and so establish a safe and effective protocol for the next generation of treatment modality, we developed a novel technique for the COMECS protocol, using a feeder-free and serum-free (FFSF) culture system. Following this new protocol, COMECS exhibited 4–5 layers of well-stratified and differentiated cells, and we successfully produced functional COMECS that included holoclone-type stem cells. Immunohistochemistry confirmed the presence of markers for cell junction (ZO1, Desmoplakin), basement membrane assembly (Collagen 7, Laminin 5), differentiation (K13, K3), proliferation (Ki67) and stem/progenitor cells (p75) in the FFSF COMECS. When transplanted to the ocular surfaces of rabbits, the tissue survived for up to 2 weeks. This study represents a first step toward assessing the development of functional FFSF COMECS for safe and ideal OSR.

Cases of severe ocular surface disease (OSD) caused by Stevens-Johnson syndrome, ocular cicatricial pemphigoid or thermal and chemical burns are potentially devastating conditions, presenting major clinical challenges. In such cases, the corneal epithelial stem cells located in the limbus are severely damaged, resulting in invasion of the corneal surface by neighboring conjunctiva, accompanied by neovascularization, chronic inflammation, fibrosis and corneal stromal scarring^1^. These conditions badly compromise ocular surface integrity and eyesight, often leading to severe OSD, afflicting many people worldwide each year^2^.

For these patients, conventional treatments such as keratoplasty have been shown to have adverse outcomes. To date, numerous clinical investigations have focused on developing surgical treatments for severe OSDs^3^,^4^,^5^,^6^,^7^, and the use of regenerative medicine and tissue engineering and cultivated epithelial stem cell transplantation has improved postoperative outcomes following ocular surface reconstruction (OSR)^8^,^9^,^10^. With others, we have demonstrated the effectiveness of autologous cultivated oral mucosal epithelial transplantation (COMET) for treatment of severe OSD, which has the advantage of reducing the risk of allograft rejection and the need for long-term immunosuppression^11^,^12^,^13^,^14^,^15^,^16^.

The currently preferred COMET method requires the use of xenobiotic materials, such as mouse-derived 3T3 feeder cells and fetal bovine serum (FBS), in the culture system. However, this raises major clinical concerns about the risk of transmission of zoonotic infection or unknown pathogens^17^. This is particularly the case when expanding cells *ex vivo* for clinical application, which increases the risk of transmission of diseases such as bovine spongiform encephalitis or other unknown infections. In light of these issues, the development of feeder-free and serum-free (FFSF) culture systems seems ideal for the next generation of COMET.

In this context, the use of human oral mucosal epithelial stem/progenitor cells in tissue-engineered cultivated grafts is another key issue, as the ability to identify stem cell populations is of great clinical value. Although we have previously reported that p75, a low-affinity neurotrophin receptor, is a potential marker for oral mucosal epithelial stem/progenitor cells^18^, there have been no reports to date regarding the development of functional cultivated oral mucosal epithelial stem/progenitor cell sheet using a defined FFSF culture system.

The purpose of the present study was to develop functional cultivated oral mucosal epithelial stem/progenitor cell sheets using a defined FFSF culture system for OSR. Our unique newly developed culture protocol enabled us to successfully generate functional cultivated oral mucosal epithelial cell sheets (COMECS) that included holoclone-type stem cells^18^,^19^,^20^. The generated cell sheets were then transplanted onto rabbit corneal surfaces, and cell sheet survival was assessed. This study represents a first step toward assessing the development of transplantable FFSF COMECS for safe and effective OSR.

## Results

### Successful Cultures of FFSF COMECS

**Figure 1:**
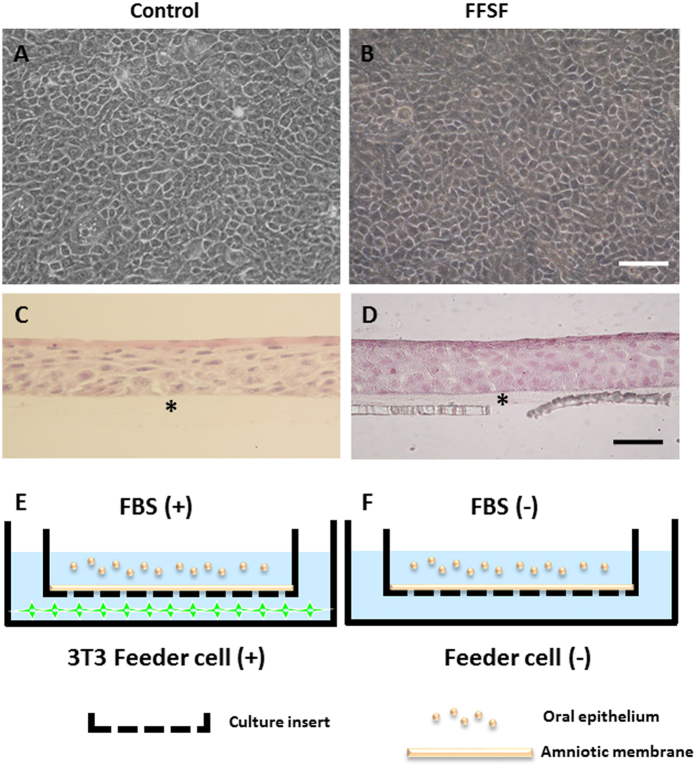
Representative phase contrast images and histological examinations of the cultivated oral mucosal epithelial cell sheet (COMECS). Phase contrast pictures showing confluent primary culture of oral mucosal epithelial cells in Control (**A**) and FFSF (**B**) after 7 days in culture; light micrographs showing cross-sections of COMECS in Control (**C**) and FFSF (**D**) culture system stained with hematoxylin and eosin; illustration of Control (**E**) and FFSF (**F**) culture conditions. Asterisks indicate denuded amniotic membrane. Scale bars = 100 μm.

Phase contrast photographs indicated that epithelial cells from the oral mucosa began to form colonies on the denuded AM within 3 days in all culture conditions (Control and FFSF). After 7 days in culture, a confluent primary culture of oral mucosal epithelial cells covered the whole AM (Fig. 1A,B). In the Control and FFSF conditions, oral mucosal epithelial cells were cobblestone-like in appearance (Fig. 1A,B). At 2 weeks in culture, both Control and FFSF COMECS exhibited 4–5 layers of stratification, were well differentiated (Fig. 1C,D) and appeared similar to normal corneal epithelium. This successfully established the FFSF COMECS protocol, which is almost similar to the previously reported culture method (Control COMECS). Culture conditions are illustrated in Fig. 1E,F.

### Proliferative Potential of FFSF COMECS

**Figure 2:**
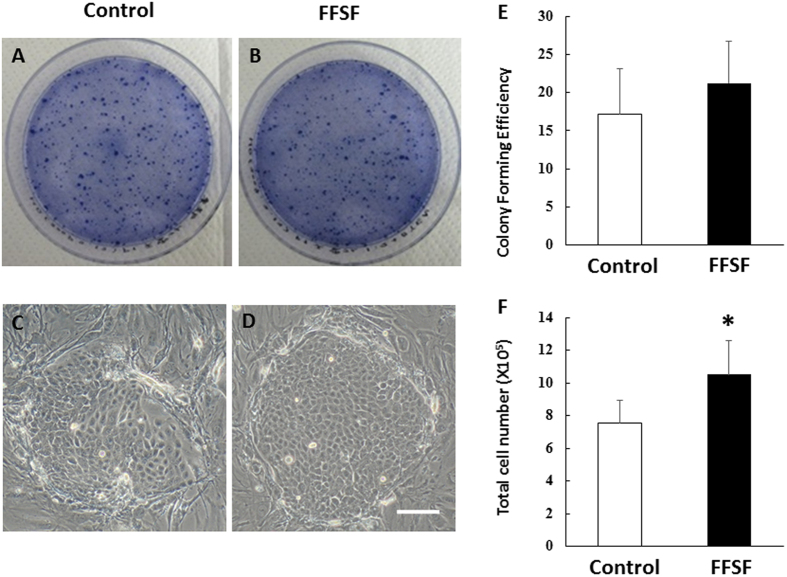
Representative colony-forming plates and phase contrast inspections of oral mucosal epithelial cells on day 7 of culture in Control (**A,C**) and FFSF (**B**,**D**) culture system. The CFE of FFSF cell conditions tended to be slightly higher than that of Control (21.2 ± 5.5% vs 17.15 ± 5.9%, respectively, N = 4) (**E**). Total cell number of FFSF cultivated oral mucosal epithelial cell sheet (COMECS) was increasing as compared to Control COMECS (10.5 ± 2.4 × 10^5^ vs 7.5 ± 1.6 × 10^5^, respectively, N = 4, *p < 0.05) (**F**).

We next examined the proliferative capacity of oral mucosal epithelial cells in Control and FFSF culture conditions using CFE assay. Phase contrast inspection of oral mucosal epithelial cells on day 7 of culture showed that both cells had formed colonies of ovoid and round cells (Fig. 2A–D). CFE tended to be slightly higher for FFSF cell conditions than for Control (21.2 ± 5.5% vs 17.15 ± 5.9%, N = 4) (Fig. 2E). We also calculated total cell number for COMECS from the same donor in both conditions and found that this was increasing as compared to Control COMECS (10.5 ± 2.1 × 10^5^ vs 7.5 ± 1.4 × 10^5^, p < 0.1, N = 4). These results indicate that the FFSF culture system maintained the proliferative potential of human oral mucosal epithelial cells as well as the previous conventional method.

### Morphological and Cell Biological Characteristics of FFSF COMECS

**Figure 3:**
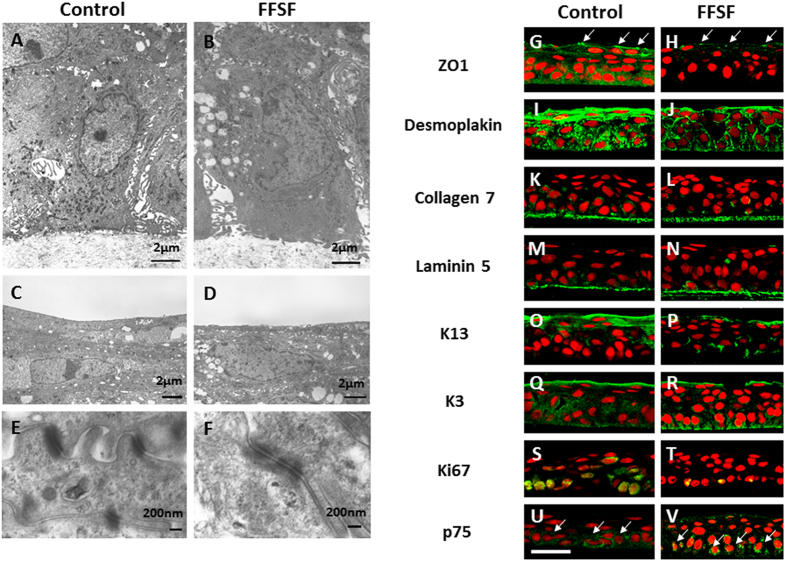
Representative morphological and cell biological features of Control and FFSF cultivated oral mucosal epithelial cell sheet (COMECS). Transmission electron microscopic examination of Control (**A,C,E**) and FFSF (**B,D,F**) COMECS; immunohistochemistry for ZO1 (**G,H**, arrows), desmoplakin (**I,J**) collagen 7 (**K,L**) laminin5 (**M,N**) K13 (**O,P**) K3 (**Q,R**) Ki67 (**S,T**) and p75 (**U,V**, arrows) in Control (**G,I,K,M,O,Q,S,U**) and FFSF (**H,J,L,N,P,R,T,V**) COMECS. Nuclei were stained with propidium iodide (red). Scale bars = 50 μm.

TEM was used to examine the morphological characteristics of FFSF COMECS. TEM examination of both Control and FFSF COMECS showed that both appeared healthy and had differentiated into basal columnar cells, suprabasal cuboid wing cells and flat squamous superficial cells (Fig. 3A–D). In all cell layers, oral mucosal epithelial cells were closely attached to neighboring cells by numerous desmosomal junctions (Fig. 3E,F).

To examine the cell biological characteristics of FFSF COMECS, immunofluorescein staining was used to identify several cell biological markers such as cell junction (ZO1, Desmoplakin), basement membrane assembly (collagen 7, laminin 5), differentiation (K13, K3), proliferation (Ki67) and stem cells (p75) (Fig. 3). ZO-1, a component of tight junction, was expressed in apical surface cells in both Control and FFSF COMECS (Fig. 3G,H). Desmoplakin, a component of cell junction, was expressed in the cell membrane of epithelium in both COMECS (Fig. 3I,J). Collagen 7 and laminin 5 were expressed in the basement membrane of both COMECS (Fig. 3K–N). Mucosal-specific keratin 13 and cornea and oral epithelial-specific keratin 3 were expressed in both COMECS (Fig. 3O–R), and we confirmed expression of Ki67, a marker of actively cycling cells, in both COMECS (Fig. 3S,T). Finally, we also found expression of p75, an oral mucosal stem/progenitor cell marker, which was clearly observed in both COMECS (Fig. 3U,V). In summary, FFSF COMECS exhibited cell junction, basement membrane, differentiation, proliferation and stem/progenitor cell phenotype characteristics considered essential for clinical adaptation and application.

### Unique Expression of Cornea-Specific Markers in FFSF COMECS

**Figure 4:**
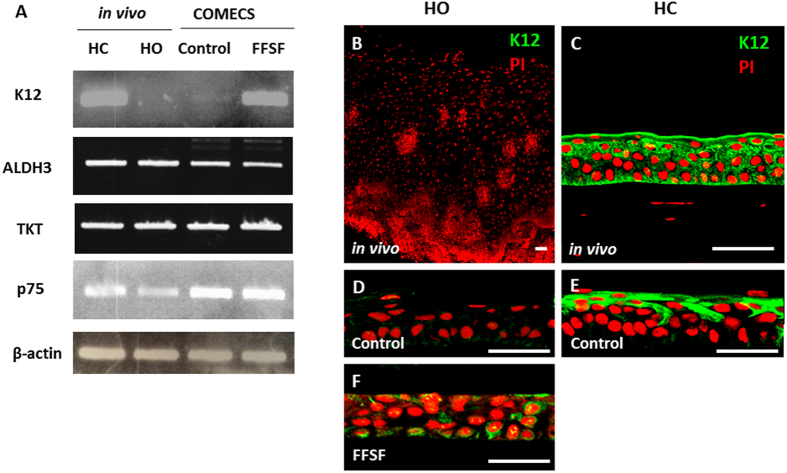
Unique expression of cornea-specific marker in FFSF cultured oral mucosal epithelial cell sheet (COMECS). Representative PCR results of the expression of cornea-specific marker (K12, ALDH3, and TKT), stem/progenitor cell marker (p75) and internal control (β-actin) (**A**). Full-length gels are presented in Supplementary Fig. 2. Immunofluorescein staining for K12 in human oral mucosal epithelium *in vivo* (**B**) Control COMECS (**D**) FFSF COMECS (**F**) human corneal epithelium *in vivo* (**C**) and cultivated corneal epithelial cell sheet (Control) (**E**); PI = propidium iodide; HC = human cornea; HO = human oral mucosa. Scale bars = 50 μm.

As it has been reported that epithelial phenotype is thought to depend largely on the surrounding environment^21^, we hypothesized that the cultivated oral mucosal epithelial cells might change their phenotype to cornea-like cells under FFSF conditions. As K12 is the most reliable marker for corneal epithelial cell phenotype among cornea-specific markers (e.g., K12, ALDH3, TKT), we examined expression of K12 in both Control and FFSF COMECS. Most interestingly, while FFSF COMECS expressed K12, Control COMECS never did (Fig. 4). Cornea markers such as ALDH3, TKT and stem/progenitor cell marker p75 were expressed in both COMECS (Fig. 4A). Examining expression of K12 using immunofluorescein staining, we found that, although neither human oral mucosal epithelium (*in vivo*) nor Control COMECS expressed K12 (Fig. 4B,D), limited sporadic expression was observed in FFSF COMECS (Fig. 4F). On this evidence, we posit that the expression pattern of K12 in FFSF COMECS somehow differs from corneal epithelium and cultivated corneal epithelial cell sheets using a conventional culture condition (Control) (Fig. 4C,E).

### Isolation and Clonal Analysis of FFSF Oral Mucosal Epithelial Stem/Progenitor Cells

**Figure 5:**
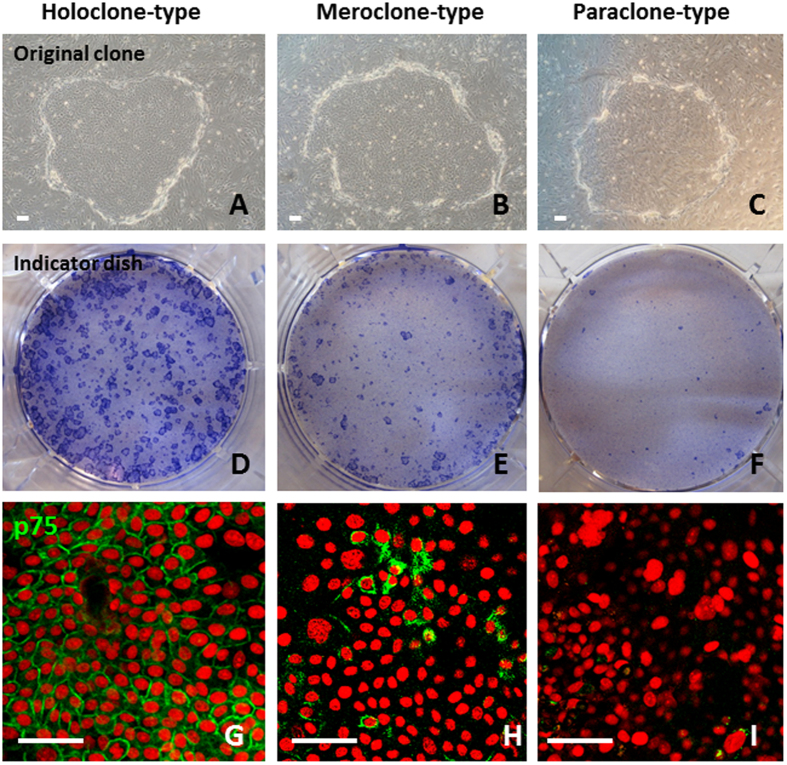
Isolation and clonal analysis of FFSF oral mucosal epithelial stem/progenitor cells. Representative original clone of the holoclone-type (**A**) meroclone-type (**B**) and paraclone-type (**C**) from FFSF cultivated oral mucosal epithelial cell sheet; toluidine blue staining (indicator dishes) of the holoclone-type (**D**) meroclone-type (**E**) and paraclone-type (**F**) cultivated oral mucosal epithelial cells; immunofluorescence staining for p75 in the holoclone-type (**G**) meroclone-type (**H**) and paraclone-type (**I**) cultivated oral mucosal epithelial cells. Nuclei were stained with propidium iodide (red). Scale bars: 50 μm.

To determine whether holoclones, meroclones and paraclones previously identified in human epithelium^19^,^20^ are also present in FFSF COMECS, we isolated single cells from FFSF COMECS obtained from 4 different donors (Supplementary Table 1). Photographs of representative holoclones, meroclones and paraclones are shown in Fig. 5. According to Barrandon and Green^19^, the representative original clone of the holoclone is large, has a smooth perimeter and contains mainly small cells (Fig. 5A); most original clones of the paraclone are small and contain large differentiated cells (Fig. 5C), and the meroclone is intermediate between the holoclone and the paraclone (Fig. 5B). On indicator dishes, holoclones formed large, rapidly growing colonies, fewer than 5% of which differentiated terminally (Fig. 5D). The paraclone grew either no colonies or uniformly small, terminal colonies (Fig. 5F), and the meroclone formed both growing and aborted colonies (Fig. 5E). Of the clones studied, 23.6 ± 12.5% were holoclones, 34.9 ± 10.5% were meroclones and 41.5 ± 11.3% were paraclones (Supplementary Table 1). Our findings indicate that holoclone-, meroclone- and paraclone-type cells previously identified in skin and ocular surfaces also constitute the proliferative compartment of FFSF COMECS.

Immunohistochemical analysis was performed to investigate expression of p75 in these three clonal types. Immunofluorescence showed that expression of p75 was clearly observed in the cell membrane of holoclone-type cells (Fig. 5G), less frequently in some of the small cells of meroclone-type cells (Fig. 5H) and rarely in paraclone-type cells (Fig. 5I). These findings confirm that our newly developed unique culture protocol can successfully generate COMECS that include p75 rich holoclone-type stem cells.

### Ocular Surface Reconstruction using FFSF COMECS

**Figure 6:**
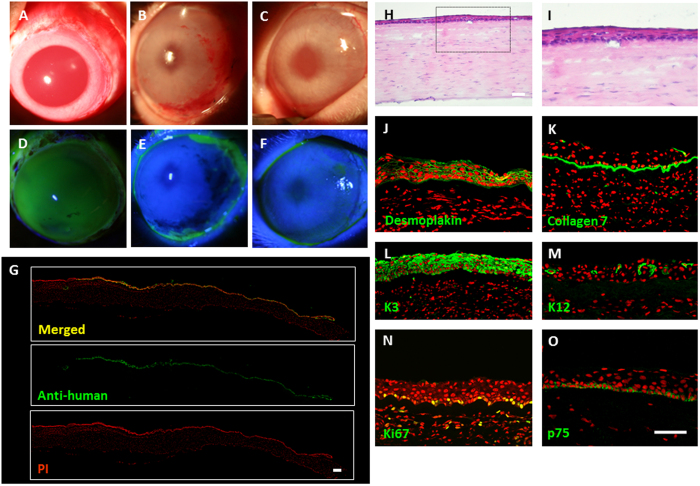
Xeno-transplantation of cultivated oral mucosal epithelial cell sheet (COMECS). Representative slit-lamp pictures of a rabbit taken just before surgery, with and without fluorescein (**A,D**) 7 days post transplantation, with and without fluorescein (**B,E**) and 14-days post transplantation, with and without fluorescein (**C,F**); immunofluorescence of anti-human nuclei at the transplanted area (**G**); representative HE staining (**H,I**) and immunofluorescence of desmoplakin (**J**) collagen 7 (**K**) keratin 3 (**L**) 12 (**M**) Ki67 (**N**) and p75 (**O**) in the transplanted FFSF COMECS. Nuclei were stained with propidium iodide (red). Scale bars = 100 μm.

Before surgery, corneal epithelial cells (including limbal region) were totally removed (Fig. 6A). Fluorescein staining confirmed the complete disappearance of corneal epithelial cells (Fig. 6D). Human FFSF COMECS were transplanted onto rabbit corneal surfaces and fixed with sutures (10–0 nylon) (N = 3). At 7 days, and again at 2 weeks following surgery, the transplanted corneas were confirmed to be clear and transparent, with no intensive inflammation (Fig. 6B,C). Fluorescein staining confirmed that the whole cornea was covered by COMECS (Fig. 6E,F). Experimental results were the same for all 3 rabbits.

Postoperative histological investigation of the COMECS revealed that they were well-attached to the host corneal stroma, with no subepithelial cell infiltrations (Fig. 6H,I). Hematoxylin-eosin staining revealed that the transplanted COMECS involved well- differentiated stratified epithelial cells (Fig. 6H,I). To certify the existence of transplanted COMECS, expression of anti-human nuclei was examined and was found to be positive in the transplanted corneal surfaces (Fig. 6G).

Expression of cell biological markers in the transplanted COMECS was then investigated. Desmoplakin was found to be expressed in the cell membrane of the COMECS (Fig. 6J). Collagen 7 was also expressed in the transplanted COMECS (Fig. 6K). K3 was clearly expressed in all transplanted COMECS, and K12 was sporadically expressed in the transplanted areas (Fig. 6L,M). Expression of Ki67 and p75 was sporadically observed in the basal cell layer of the COMECS (Fig. 6N,O). It was concluded that transplanted cells on the rabbit corneal surface still maintain proliferative and stem/progenitor cell properties. Two weeks after transplantation, human oral mucosal epithelial cells were isolated from the transplanted rabbit corneal surface. These cells showed colony-forming ability, with small cuboidal cells and expression of p75, Ki67, K15 and K12 (Supplementary Fig. 1A–F). These results indicate that the FFSF COMECS is functionally well-adapted to *in vivo* environments.

### Gene Expression Profile of FFSF COMECS

**Figure 7:**
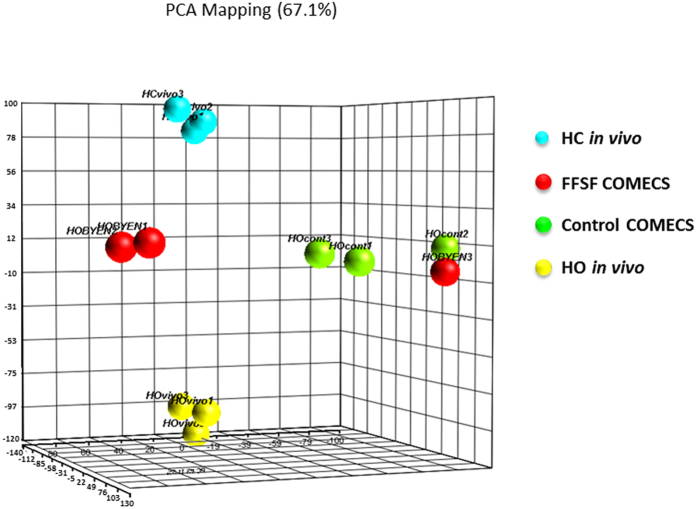
Gene expression profile of FFSF cultivated oral mucosal epithelial cell sheet (COMECS). Principal component analysis (PCA) mapping of human corneal epithelium (HC) *in vivo*, human oral mucosal epithelium (HO) *in vivo*, Control COMECS and FFSF COMECS (N = 3).

To gain some insight into the molecular mechanisms responsible for the effect of the FFSF condition, gene expression profiling of Control and FFSF COMECS was performed, using DNA chip analysis. By way of comparison, tissue human corneal and oral mucosal epithelial cells were also investigated. Principal component analysis (PCA) mapping indicated that gene expression profiles of human corneal epithelium *in vivo* were quite different from those of human oral mucosal epithelium *in vivo* (Fig. 7). Interestingly, the gene expression profiles of both Control and FFSF COMECS were intermediate between human corneal and oral mucosal epithelium *in vivo*, and those of FFSF COMECS tended to be closer to human corneal epithelium *in vivo* than those of Control COMECS, suggesting that the FFSF culture system may influence the integrity and characteristics of oral mucosal epithelial cells.

## Discussion

Severe OSD currently poses a serious clinical challenge for ophthalmologists worldwide. OSR using COMET is a newly developed regenerative medicine that promises to advance therapeutic modalities^11^,^12^,^13^,^14^,^15^,^16^. Previous studies on COMET have relied primarily on xenobiotic materials such as FBS and 3T3 cells, and the risk of transmission of zoonotic infection or unknown pathogens is a major clinical concern. The use of a FFSF culture system for the development of tissue-engineered ocular surface equivalents would be of particular clinical relevance in such cases. The present findings confirm for the first time the successful generation of “functional” COMECS that include p75 (+) holoclone-type stem/progenitor cells, using our newly developed culture protocol.

The development of a FFSF culture system has long been a challenge for scientists worldwide. Yokoo *et al*.^22^ previously reported development of human cultivated corneal epithelial sheets using a defined FFSF culture system containing EGF and B-27. As corneal and oral mucosal epithelial cells differ in cell character, their responses to this culture system also differed somewhat. In determining a culture protocol, we finally established that a combination of EGF, Y-27632, B27, hydrocortisone, epigallocatechine gallate and dextran 40 is better in generating the functional cultivated oral mucosal epithelial stem/progenitor cell sheet. Although B27 contains purified albumin derived from prion-free New Zealand cows, these findings bring us a step closer to finding a safe and effective FFSF bioengineered tissue equivalent for clinical transplantation. In future stages of the developmental process, we will use a human recombinant albumin as a substitute.

At a practical level, the main advantages of our FFSF culture system are its stability and minimal variability of culture procedure. Using FBS- and MMC-treated 3T3 feeder cells (Control), we commonly found that variations in FBS and 3T3 cell conditions undermined culture results. Our FFSF system does not depend on these variable factors; we found that total cell number in FFSF COMECS increased as compared to Control COMECS, and FFSF COMECS clearly showed the expression of markers for actively cycling cells (Ki67). This suggests that the FFSF culture system maintained the proliferative potential of human oral mucosal epithelial cells.

With regard to the feasibility and efficacy of using FFSF COMECS for successful transplantation, it was important to elucidate how well the cultured sheet maintained the morphological and cell biological character of the functional epithelium. Morphological and immunofluorescence investigations indicated that the proteins related to cell junction and basement membrane were clearly expressed in the FFSF COMECS, where adjacent cells were connected by multiple desmosomal junctions. Additionally, tight junctions were observed between the most superficial cells. These results encouraged us to transplant the FFSF COMECS onto an *in vivo* corneal surface.

The epithelial phenotype is thought to depend on the surrounding environment and stem cell niche. In our experiments, we were surprised to find that FFSF COMECS showed expression of K12 at both mRNA and protein levels. Although its mRNA expression level was much lower than *in vivo* corneal epithelium, and protein expression of K12 was detected only in small restricted areas, the FFSF culture system may well influence epithelial lineage. Based on our results, including PCR, immunohistochemistry and gene expression profile, we are unable to determine whether cultivated oral epithelial cells can transdifferentiate into a cornea-like cell lineage, and further work is underway in our laboratory to clarify this observation.

To increase the probability of long-term corneal regeneration, it is important that stem/progenitor cells are retained in the cultivated oral mucosal epithelial cell sheet, as these cells have greater proliferative potential. We have previously demonstrated that p75 identified oral mucosal epithelial stem/progenitor cells^18^, and expression of p75 was clearly observed in both the FFSF COMECS and transplanted FFSF COMECS. Our single cell-based clonal analysis indicated that the FFSF culture medium maintained p75 (+) holoclone-type oral mucosal stem cells. These findings suggest that our newly developed FFSF culture system was able to maintain and perhaps even support the proliferation of stem/progenitor cells.

Following successful cultivation, FFSF COMECS was transplanted onto corneal surfaces to examine its viability. Fourteen days after xeno-transplantation of FFSF COMECS, the corneal surfaces were free of epithelial damage, suggesting that the FFSF COMECS had survived. The presence and proliferation of junctional proteins and stem/progenitor cell markers indicate that the FFSF COMECS has the potential to maintain corneal integrity and to treat severe OSD.

In conclusion, this study is the first to elucidate the survival of tissue-engineered FFSF COMECS. Using our newly developed culture system, we successfully generated functional cultivated oral mucosal epithelial stem/progenitor cell sheets. We then successfully transplanted FFSF COMECS onto rabbit corneal surfaces, and we are convinced that this surgical approach to the treatment of severe OSD has great potential. As the long-term outcome of COMET using the FFSF culture system remains unclear, further investigations are needed to clarify its applicability in clinical situations.

## Materials and Methods

### Tissues

All methods were designed according to the Declaration of Helsinki’s ethical principles. All of the experimental protocols described here were approved by the Institutional Review Board for Human Studies of Kyoto Prefectural University of Medicine, Kyoto, Japan (Approval No. RBMR-R-20). Prior informed consent was obtained from all participants in accordance with the Declaration of Helsinki for research involving human subjects. Oral tissues were obtained from healthy volunteers and as superfluous tissue from patients undergoing oral surgery. All samples were processed within 1–2 hours of harvest.

### Cell Culture

Human oral mucosal epithelium was cultured, following a previously reported method^11^,^12^,^15^. Briefly, oral mucosal biopsy was performed using local anesthesia. The oral mucosa was then incubated at 4 °C for 5–6 hours with Dispase (1.2 IU), followed by treatment with Trypsin-EDTA solution (0.05%) for 10 minutes to separate the epithelial cells. The resultant oral mucosal epithelial cells (1–2 × 10^5^ cell/ml) were then disseminated onto denuded amniotic membrane (AM), distributed on the basis of cell culture inserts. The FFSF culture medium consisted of DMEM/F12 (Life Technologies Corporation, Carlsbad, CA), defined keratinocyte-SFM (Life Technologies) (mixture ratio 1:2) with recombinant human EGF (10ng/ml) (Life Technologies), Rho-associated protein kinase (ROCK) inhibitor Y-27632 (10 μM/L) (Abcam PLC, Cambridge, MA), B27 (2%) (Life Technologies), hydrocortisone (1 μg/ml) (Lonza), (−)-epigallocatechin gallate (10 mg/ml) (Sigma-Aldrich, St. Louis, MO), Dextran 40 (1%) (Tokyo Kasei Kougyou, Tokyo) and penicillin-streptomycin (50IU/ml) (Life Technologies). As controls, we used keratinocyte growth medium (KGM: ArBlast Co., Ltd., Kobe, Japan) supplemented with 5% FBS (Hyclone, Tauranga, New Zealand), co-cultured with mitomycin C (MMC)-inactivated NIH-3T3 fibroblasts (specifying this condition as “Control”). The cultured cells in each condition were submerged in medium for 2 weeks and then air-lifted for 1–2 days by lowering the medium level. The schema for each cell culture condition is illustrated in Fig. 1(G–I).

For human corneal epithelial cell cultivation, donor corneas (SightLife,^®^ Seattle, WA) were initially incubated at 37 °C for 1 hour with Dispase (1.2 IU) to dissociate the corneal epithelial cells. The resultant corneal epithelial cells were then disseminated onto denuded AM, distributed on the basis of cell culture inserts, and co-cultured with MMC-inactivated NIH-3T3 feeder cells.

### Colony-forming efficiency

The clonal growth ability of each COMECS (Control and FFSF) was determined by colony-forming efficiency (CFE) assay. Cells (2 × 10^3^) from each COMECS were plated on 6-well culture dishes containing a feeder layer of MMC-inactivated NIH-3T3 fibroblasts (N = 4). Colonies were fixed on day 7, stained with 0.1% toluidine blue, and independently counted by three investigators, and the data were then averaged. CFE was defined as the ratio of number of colonies to number of viable cells seeded.

### Examination by Transmission Electron Microscopy

Samples from each COMECS (Control and FFSF) were examined by transmission electron microscopy (TEM). Samples were immersed in 2.5% glutaraldehyde in 0.1 M phosphate-buffered saline (PBS). After glutaraldehyde fixation they were given three 15 minute washes in PBS. Following this they were placed in 2% aqueous osmium tetroxide for 2 hours, after which they were given three 15 minute washes in PBS. Following this the samples were dehydrated through a series of ethanol solutions (50, 70, 80, 90, 95 and 100%). For TEM the samples were embedded in araldite resin (Agar Scientific Ltd., Stansted, UK); sections 70 nm thick were cut with a diamond knife and collected onto 300 mesh copper grids. The grids were stained for 1 hour with uranyl acetate, washed in distilled water then stained for 30 minutes with 1% phosphotungstic acid, washed with distilled water and then stained for 20 minutes with Reynolds’ lead citrate. The grids were then examined in a JEM 1010 TEM (JEOL Ltd., Tokyo, Japan).

### Antibodies and Reagents

For immunofluorescence staining, the following antibodies (Abs) were used: mouse monoclonal Abs anti-keratin 13 (x200) (Novocastra Ltd., Newcastle upon Tyne, UK), anti-keratin 3 (x50) (Progen Biotechnik GmbH, Heidelberg, Germany), anti-keratin 15 (x50) (Abcam), anti-ZO1 (x25) (Zymed Laboratories, Inc., South San Francisco, CA), anti-desmoplakin (x1) (Progen Biotechnik), anti-laminin5 (x100) (Chemicon International, Inc., Temecula, CA), anti-collagen 7 (x100) (Chemicon International), anti-p75 (x200) (Abcam), anti-Ki67 (x200) (BD Pharmingen), anti-human (X100) (Chemicon International) and rabbit polyclonal Abs anti-keratin 12 (x200) (TransGenic Inc., Kumamoto, Japan). Secondary Abs contained Alexa 488 goat anti-mouse or rabbit IgG (x1500) (Molecular Probes, Inc., Eugene, OR).

### Immunofluorescence staining

Immunofluorescence staining followed our previously described method^11^,^12^,^15^,^18^,^20^. Briefly, cryostat sections (8–10 *μ*m) on silane-coated slides or cultured cells in chamber slides (Iwaki Glass Co., Ltd., Chiba, Japan) were air-dried, rehydrated in PBS for 20 minutes, and fixed in 100% Acetone at 4 °C for 15 minutes. Next, the slides were washed with PBS containing 0.15% TRITON™ X-100 surfactant (Dow Chemical Company, Midland, MI) at room temperature (RT = 24 °C) for 15 minutes. To block nonspecific binding, the slides were incubated with 1% bovine serum albumin at RT for 30 minutes. Subsequently, slides were incubated with primary Abs at RT for 1 hour and washed 3 times in PBS containing 0.15% TRITON™ X-100 for 10–15 minutes. The normal mouse and rabbit IgG (at the same concentration as the primary antibody) were used as isotype-matched control. Slides were then incubated with the appropriate secondary Abs at RT for 1 hour. After several washings with PBS, the slides were then coverslipped with glycerol involving propidium iodide (PI) (Nacalai Tesque, Inc., Kyoto, Japan). Finally, these were investigated using a confocal microscope (FluoView^™^; Olympus Corporation, Tokyo, Japan).

### Polymerase Chain Reaction (PCR)

PCR was performed following our previously described method^18^,^20^. All samples were homogenized in a lysis buffer (Buffer RLT; QIAGEN, Inc., Valencia, CA), and total RNA was eluted using the RNeasy^®^ Mini Kit (QIAGEN) according to the manufacturer’s instructions. Complementary DNA (cDNA) was generated by mixing extracted RNA (1 μg/μl/sample) with a random hexamer primer (Takara Biomedicals, Tokyo, Japan) and AMV Reverse Transcriptase (RT) XL (Takara Biomedicals). The PCR conditions were 30 seconds denaturation at 96 °C, 30 seconds annealing at 55 °C, and 40 seconds elongation at 72 °C (35 cycles). PCR products were size-fractionated by 1% agarose gel electrophoresis. The primers used are shown in Supplemental Table 2.

### Clonal analysis

For clonal analysis, we applied the method of Barrandon and Green^18^,^19^,^20^, using secondary cultures of human oral mucosal epithelial cells and the FFSF system. Briefly, we first prepared culture medium, including dissociated oral epithelial cells (cell suspension), in a 10 cm culture dish. This was placed on the inverted microscope. We then set up the microchip in the culture medium, and we directly observed that a single cell was aspirated using a micropipette. In this way, single cells were isolated under an inverted microscope and inoculated into 12-well plates containing a feeder layer of MMC-inactivated NIH-3T3 cells. After 7 days, a single clone was identified under an inverted microscope and photographed. Each clone was then divided into 2 parts, of which three-quarters was subjected to immunocytochemical analysis to evaluate cell characteristics. The other quarter of the clone was transferred to an indicator dish, fixed 10–12 days later, and stained with 0.1% toluidine blue for classification of clonal type, as determined by the percentage of aborted colonies. Where 0–5% of colonies were terminal, the clone was classified as a holoclone; where all colonies were terminal or where no colonies formed, the clone was classified as a paraclone; and where more than 5% but less than 100% of colonies were terminal, the clone was classified as a meroclone.

### Xeno-COMET

Following our established protocol^23^, the human FFSF COMECS were transplanted to white rabbits (2–2.5 kg). General anesthesia was performed by intramuscular injection of xylazine hydrochloride (5 mg/mL) and ketamine hydrochloride (50 mg/mL). All animals were handled with due regard to the ARVO Statement for the Use of Animals in Ophthalmic and Vision Research. The experiments were approved by the Animal Research Committee at Kyoto Prefectural University of Medicine.

An ocular surface injury was created in the adult albino rabbits by excising all conjunctival tissue within 5 mm of the limbus, performing a superficial keratectomy of the entire corneal surface, including the limbal epithelial cells. Then, human COMECSs were transplanted onto the keratectomized corneal surface with 10–0 nylon sutures. Following transplantation, the rabbits were intramuscularly administered FK506 (300 μ/day) (Astellas Pharma Inc., Tokyo, Japan) for 2 weeks and treated each day with antibiotic and steroid ointment (Santen Pharmaceutical Co., Ltd, Osaka, Japan & Shionogi Co., Ltd., Osaka, Japan).

### Gene expression analysis

Gene expression profiles were examined by use of a high-density oligonucleotide probe array, GeneChip Human Genome U133 Plus 2.0 (Affymetrix, Santa Clara, CA)^20^. Total RNA was extracted using the RNeasy^®^ kit (Qiagen, Venlo, Netherlands). cRNA preparation and target hybridization were performed according to the Affymetrix GeneChip technical protocol. The DNA chips were scanned using a specially designed confocal scanner (GeneChip Scanner 3000; Affymetrix). Array data analysis was performed with Affymetrix GeneChip operating software (GCOS) version 1.0.

## Additional Information

**How to cite this article**: Nakamura, T. *et al*. Development of functional human oral mucosal epithelial stem/progenitor cell sheets using a feeder-free and serum-free culture system for ocular surface reconstruction. *Sci. Rep.* **6**, 37173; doi: 10.1038/srep37173 (2016).

**Publisher’s note**: Springer Nature remains neutral with regard to jurisdictional claims in published maps and institutional affiliations.

## Supplementary Material

Supplementary Information
