# Supplementary material for: Development of functional human oral mucosal epithelial stem/progenitor cell sheets using a feeder-free and serum-free culture system for ocular surface reconstruction
Source: Sci Rep. 2016 Nov 14;6:37173. doi: 10.1038/srep37173 (PMC5107917; doi:10.1038/srep37173)
Supplement: Supplementary Information [file srep37173-s1.pdf]

Supplementary Information

Development of functional human oral mucosal epithelial stem/progenitor cell sheets using a feeder-free and serum-free culture system for ocular surface reconstruction

Takahiro Nakamura, Seiichi Yokoo, Adams J Bentley, Maho Nagata, Nigel J. Fullwood, Tsutomu Inatomi, Chie Sotozono, Satoru Yamagami, Shigeru Kinoshita

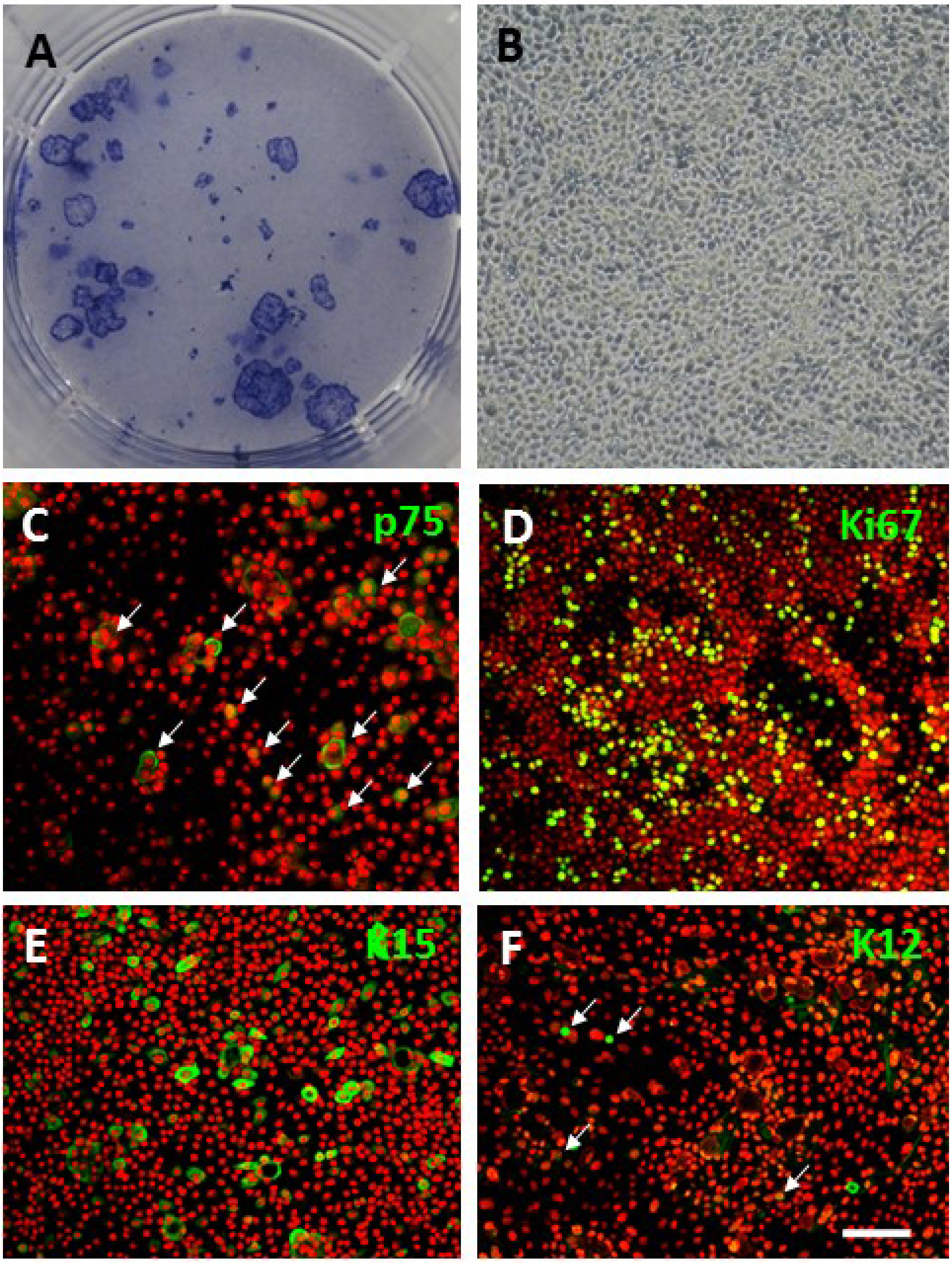

Supplementary Figure 1. Characterization of transplanted FFSF cultivated oral mucosal epithelial cell sheet (COMECS). Colony forming ability of transplanted FFSF COMECS (A). Phase contrast image of transplanted cells (B). Immunofluorescein staining of p75 (C, arrows), Ki67 (D), K15 (E) and K12 (F, arrows) in transplanted cultivated oral mucosal epithelial cells. Nuclei were stained with propidium iodide (red). Scale bars = 100μm.

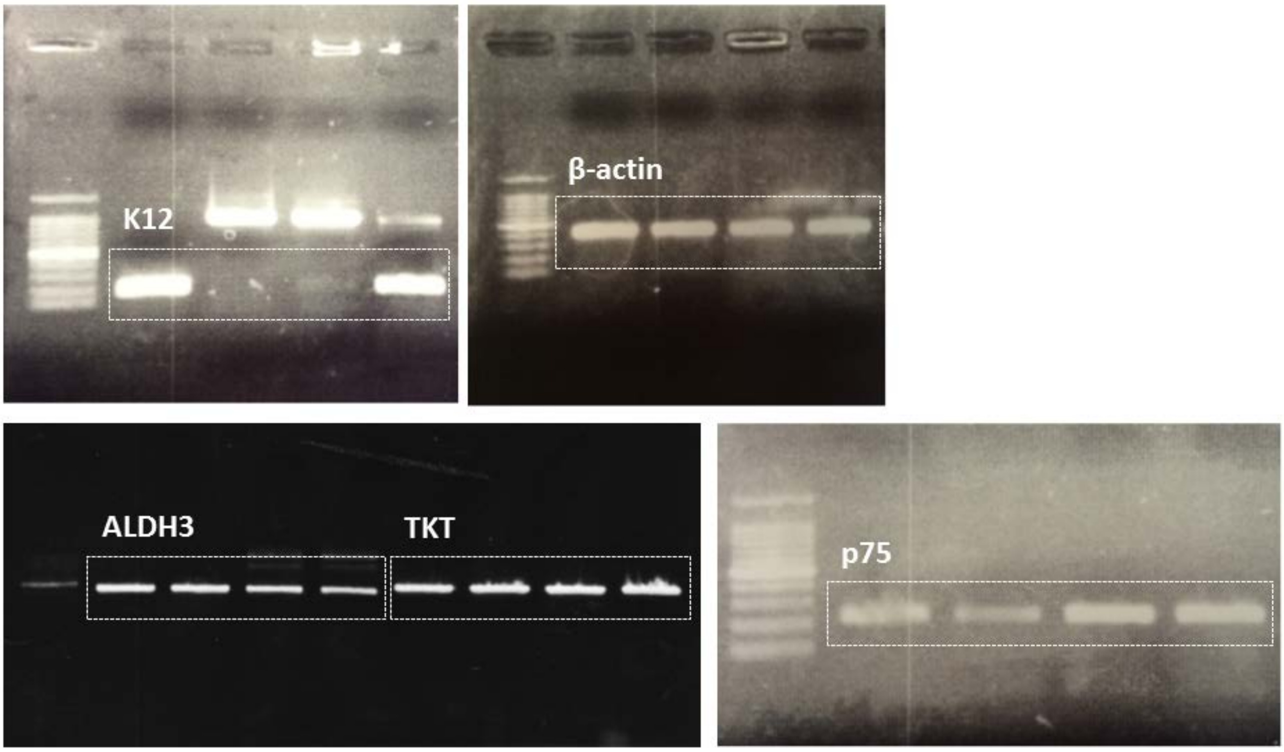

Supplementary Figure 2. Full-length gels of Figure 4A.

Supplementary Table 1. Classification of clonal types

Donor age/gender	Each type of clone(%)		
	Holoclone	Meroclone	Paraclone
30/M	28.6%	28.6%	42.8%
27/M	16.7%	16.7%	66.6%
38/M	33.3%	25%	41.7%
37/F	4.2%	45.8%	50%

Supplementary Table 2. Sequences for PCR

human K12	F	AATCATGGGGCAGATCTTGT
	R	AAGGTGATGGTTTGGAGGAA
human ALDH3	F	TTGCAGAGACATCCAGTGGT
	R	TTGGTCTAGAAAGGGGTGGA
human TKT	F	CTGCTTCATCCGGACCAG
	R	CACACTTCATACCCGCCCTA
human p75	F	TGAGTGCTGCAAAGCCTGCAA
	R	TCTCATCCTGGTAGTAGCCGT
human β-actin	F	GGACTTCGAGCAAGAGATGG
	R	ATCTGCTGGAAGGTGGACAG

F: Forward, R: Reverse
